# Beyond Chemistry: Tailoring Stiffness and Microarchitecture to Engineer Highly Sensitive Biphasic Elastomeric Piezoresistive Sensors

**DOI:** 10.1021/acsami.2c04673

**Published:** 2022-04-22

**Authors:** Matteo Solazzo, Linette Hartzell, Ciara O’Farrell, Michael G. Monaghan

**Affiliations:** † Department of Mechanical, Manufacturing and Biomedical Engineering, Trinity College Dublin, Dublin 2, Ireland; ‡ Trinity Centre for Biomedical Engineering, Trinity College Dublin, Dublin 2, Ireland; § Advance Materials and BioEngineering Research (AMBER) Centre at Trinity College Dublin and the Royal College of Surgeons in Ireland, Dublin 2, Ireland; ∥ CÚRAM, Centre for Research in Medical Devices, National University of Ireland, Galway, Newcastle Road, H91 W2TY Galway, Ireland

**Keywords:** piezoresistive sensors, conductive polymers, pedot:pss, strain sensors, conductive elastomer

## Abstract

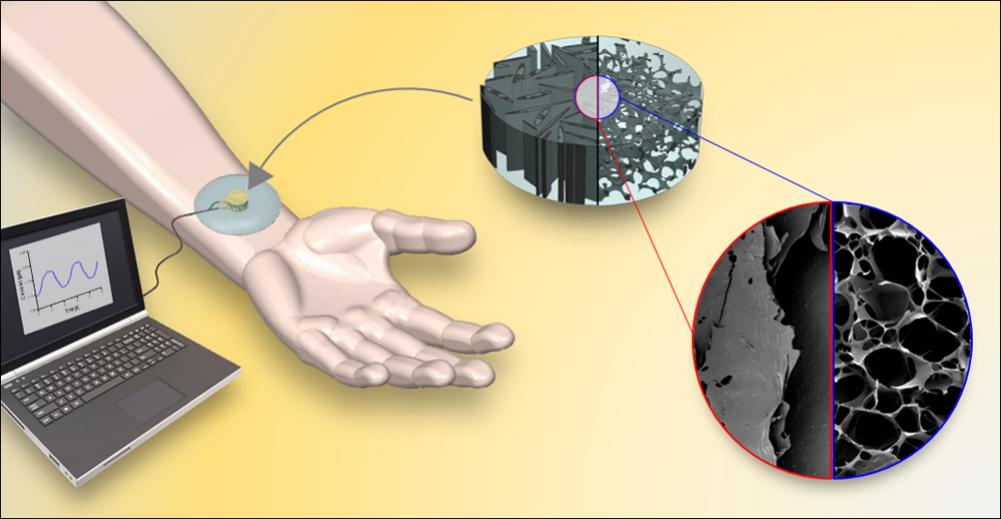

Carbon-based nanoparticles and conductive polymers are two classes of materials widely used in the production of three-dimensional (3D) piezoresistive sensors. One conductive polymer, poly(3,4-ethylenedioxythiophene):polystyrenesulfonate (PEDOT:PSS) has excellent stability and conductivity yet is limited in its application as a sensor, often existing upon a base, limiting its performance and potential. Despite much progress in the field of materials chemistry and polymer synthesis, one aspect we consider worthy of exploration is the impact that microstructure and stiffness may have on the sensitivity of 3D sensors. In this study, we report a strategy for fabricating biphasic electroactive sponges (EAS) that combine 3D porous PEDOT:PSS scaffolds possessing either an isotropic or anisotropic microarchitecture, infused with insulating elastomeric fillers of varying stiffness. When characterizing the electromechanical behavior of these EAS, a higher stiffness yields a higher strain gauge factor, with values as high as 387 for an isotropic microarchitecture infused with a stiff elastomer. The approach we describe is cost-effective and extremely versatile, by which one can fabricate piezoresistive sensors with adaptable sensitivity ranges and excellent high strain gauge factor with the underlying microarchitecture and insulant stiffness dictating this performance.

## 1 Introduction

Wearable sensor technology has seen dramatic growth in demand in recent years with a range of applications as affordable and personalized diagnostic and continuous monitoring tools within medicine,^1,2^ fitness, as well as within robotics for electronic skin^3^ and tactile sensing.^4^ Such sensors can aid in early detection of acute health deterioration in a hospital setting^5^ or provide long-term monitoring in a patient’s home.^6^ A wide range of technologies have been developed and implemented, among which piezoresistive sensors are frequently used due to their high sensitivity, simple device structure, and easy to interpret readout.^7^

Mechanical deformation of a piezoresistive material, emanating from applied stress or strain, causes transduction of the mechanical force into a change in piezoresistivity and material conductivity, known as the piezoresistive effect.^8^ The performance of a piezoresistive material as a wearable strain sensor is based on certain criteria, including low hysteresis, high sensitivity, compressibility, and electromechanical stability under repeated dynamic loading conditions.^9,10^ Piezoresistive materials can also be used within pressure sensors, which have applications in detection of subtle pressures such as blood flow or “touch” in the context of, for example, brain machine interfaces.^11^

Conductive polymers have emerged as candidate sensor materials^12^ with chemical, mechanical, and electrical properties that can be tailored toward specific applications by virtue of the method by which they are manufactured as well as through various treatments.^13,14^ Poly(3,4-ethylenedioxythiophene):polystyrenesulfonate (PEDOT:PSS) is a conjugated polymer with high chemical and environmental stability,^15,16^ which has already seen many applications within the field of wearable sensors.^17−22^ It consists of the highly conductive PEDOT combined with insulating PSS, which acts as a counterion to PEDOT to stabilize its chemical properties. When combined, they exist as a semiconductive compound with piezoresistive properties which could be utilized for the purpose of a strain or pressure piezoresistive sensor. A drawback to PEDOT:PSS lies in its relatively brittle nature as it can only be stretched to about 10% without plastic deformation,^23^ and its proneness for delamination and redispersion in aqueous or humid environments. Cross-linking is a common method used in the context of enhancing stability of PEDOT:PSS in aqueous environments with glycidoxipropyl-trimethoxysilane (GOPS) being an extensively used option.^16,24^

A balance between stretchability, sensitivity and linearity is a common challenge in piezoresistive sensors, with stretchability limiting sensitivity in both low strain or pressure ranges as well as causing increased mechanical and electrical hysteresis through the viscoelastic properties of polymers, which limits the use of piezoresistive sensors in the realm of dynamic high-frequency measurements. A porous or foam three-dimensional (3D) structure can have improved stretchability and linearity of both the mechanical and electrical behavior^25^ which increases their applicability for dynamic loading conditions. Porous piezoresistive sensors to optimize sensor behavior across a wide range of stresses and strains have therefore frequently been explored in recent years.^18,26−29^ The use of optimized microstructures such as pyramids or biomimetic textures have been shown to enhance sensor performance further.^27,30,31^ 3D porous PEDOT:PSS scaffolds can be produced through lyophilization, during which pore size and alignment can be controlled through predefined parameters.^16^ Although lyophilized PEDOT:PSS scaffolds are previously reported,^32,33^ their electromechanical response and the applicability for piezoresistive sensor applications have never been explored.

One common approach for the fabrication of piezoresistive sensors consists in the introduction of conductive particles in an insulating polymer matrix and attaining conductive composites. Among such insulating matrices, polydimethylsiloxane (PDMS) is a silicone rubber elastomer unique in its high flexibility, along with high compressibility, wide operational temperature range, and nontoxicity.^34^ It can easily be handled in the laboratory and is one of the most frequently incorporated materials in flexible sensors,^35^ where its elastic behavior offers the benefit of low mechanical drift and low hysteresis under cyclic loading.^36^ When combined with conductive infills such as graphene,^26,37^ carbon nanotubes (CNTs),^37,38^ carbon black,^39^ or silver nanoparticles,^40^ they form highly conductive nanocomposites with applications as piezoresistive sensors. Moreover, PDMS can be found in different chemical forms, and this makes it possible to achieve final compounds with diverse mechanical properties by simple combination of these forms at specific ratios.^41^

Although many reports describe piezoresistive materials with remarkably high sensitivity due to their chemistry, the impact of microarchitecture and the material stiffness on the performance of piezoresistive materials has only recently been investigated^42^ and mostly limited to analysis of the size of the pores in foamlike structures,^43^ and rarely to the orientation of the pores.^44^

**Figure 1:**
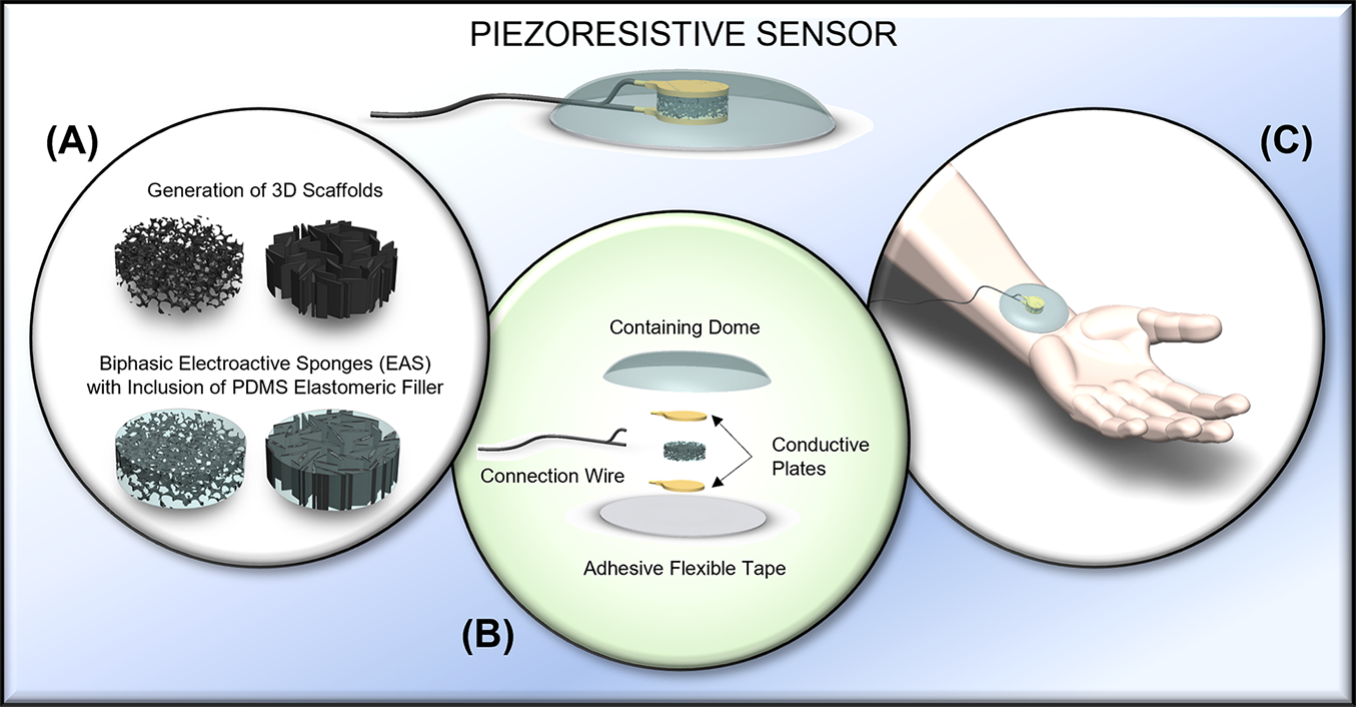
Conceptualization of the PDMS-embedded PEDOT:PSS-based piezoresistive sensor. (A) Illustrations of the generation of 3D PEDOT:PSS-GOPS scaffolds with both isotropic and aligned microarchitecture, followed by the inclusion of a PDMS elastomeric filler to generate a biphasic EAS. (B) Schematic of the assembly of the piezoresistive sensor showing the components: a containing dome, two conductive plates, one EAS, an adhesive flexible tape, and a connection wire. (C) Simulation of the application of the piezoresistive sensor to a body part for recording of physiological signals.

Here, we describe the electromechanical behavior of porous, microstructured PEDOT:PSS-based electroactive sponges (EAS) and demonstrate their application as piezoresistive sensors for health monitoring. After predesigning EAS to possess either isotropic or aligned architectures, we embedded them within PDMS insulant elastomeric matrices characterized of varying stiffnesses (Figure 1A). This strategy facilitates biphasic EAS of different mechanical properties while maintaining the original morphology of the PEDOT:PSS-based scaffolds. These structures were introduced as sensor modules within a prototype sensor (Figure 1B), where we demonstrate that the material has the capability to measure a variety of physiological signals, including swallowing, muscle tensing, and limb movements including finger and knee bending (Figure 1C).

## 2 Results and Discussion

### 2.1 Fabrication of Biphasic Electroactive Sponges (EAS)

Lyophilization was employed to generate highly porous structures of PEDOT:PSS cross-linked using GOPS as reported previously.^15,16^ This lyophilization approach is a scalable process and facilitates constructs of different sizes and shape.

#### 2.1.1 Processing of PEDOT:PSS-GOPS Scaffolds and Morphological Analysis

Various microstructures have been proposed to enhance sensor performance, such as pinnate-veined pores created through freeze-drying,^45^ or biomimetically textured materials.^46^ One previous study has looked to improve mechanical recovery of the foam under dynamic compression by fabricating an aligned CNT-thermoplastic PU material through directional freeze-drying, which exhibited excellent linear recovery in comparison to a disordered foam.^44^

**Figure 2:**
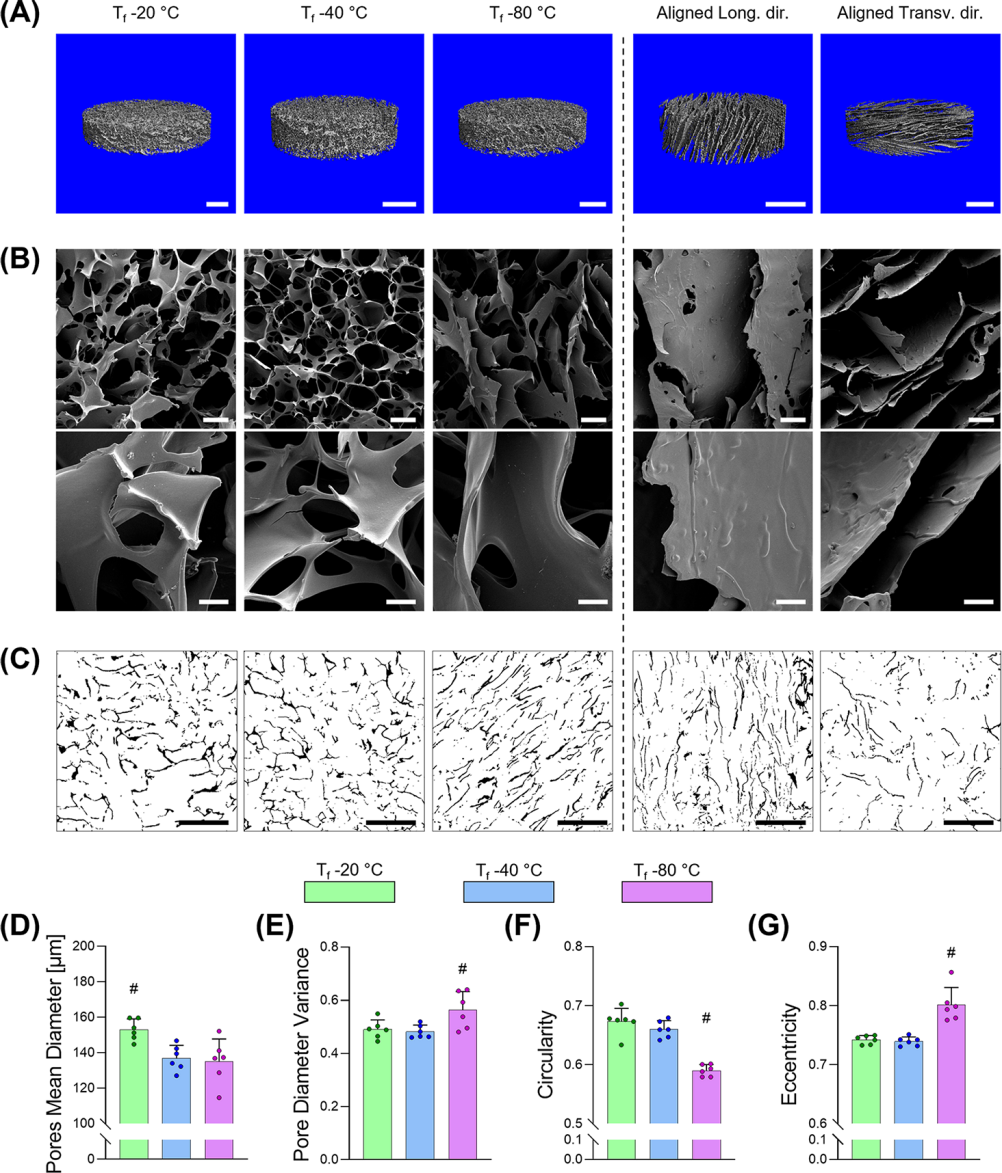
Morphological characterization of EAS. (A–C) Image analysis of isotropic samples processed at different *T*_f_, namely, −20 °C, −40 °C, and −80 °C and aligned samples observed along the longitudinal and the transversal directions with different techniques: (A) microCT, (B) SEM micrographs at different magnifications, (C) sliced agarose embedded scaffolds. (D–G) Quantitative analysis of the effect of *T*_f_ on the size and shape of isotropic samples (*n* = 3 per direction per sample): (D) pores mean diameter, (E) pores mean variance, (F) circularity, and (G) eccentricity. Scale bars: (A) = 1 mm; (B, top) = 100 μm; (B, bottom) = 20 μm; (C) = 500 μm. Bar graphs demonstrate the mean with error bars representing standard deviation. Data values are presented as associated points. # represents statistical significance (*p* < 0.05) between the indicated group and all other groups using one-way ANOVA with Tukey’s posthoc test.

In this study, we investigate to what extent the microarchitecture of an electroconductive sensor can influence the overall piezoresistive response. In this respect, we compared PEDOT:PSS-GOPS sponges that were designed to possess an isotropic or anisotropic structure. In our previous work,^16^ the adoption of standard plastic cell culture well plate and of custom-made molds (Figure S1) yielded the fabrication of both isotropic and anisotropic architectures (Figure 2).

We first investigated the generation of isotropic sponges with the most homogeneous porosity and circularity of the pores for a relevant comparison with the highly aligned microarchitecture typical of the anisotropic scaffolds. It is well established that freezing temperature (*T*_f_) affects the pore microarchitecture; in particular, the wider the difference between the temperature of the material and the *T*_f_ is, the faster the nucleation rate of the ice crystals and the slower the heat transfer from the nucleation site are, ultimately causing smaller crystals and a reduction in pore size.^47^ In addition, general cooling condition, such as cooling rate, determines the shape of the ice crystals which can be described by their circularity and eccentricity.^48^

Here, we applied three different *T*_f_ to produce PEDOT:PSS-GOPS scaffolds, namely −20 °C, −40 °C, and −80 °C. This was achieved by placing samples directly into a precooled freezing chamber. In this manner, although the cooling rate was not predefined, as in the case of a stepwise control, the thermal difference between the solutions and the freezing chamber indirectly generated different cooling rates. Although they have not been defined, such cooling rates were consistent, guaranteeing reproducibility of the manufactured scaffolds which was reflected through all analyses. From an initial macroscale observation using microCT (Figure 2A), no major pore differences were appreciable between the three processing conditions; however, SEM micrographs detected a more irregular porous architecture in the −80 °C group (Figure 2B). This was confirmed by a quantitative analysis on sectioned samples through which a series of parameters were determined (Figure 2C). Specifically, a statistically significant increase in pore size was present at a *T*_f_ of −20 °C; an average pore diameter of 153 ± 6 μm was present when compared with the 137 ± 7 and 135 ± 13 μm of the −40 °C and −80 °C, respectively (Figure 2D). Scaffolds processed at −80 °C showed significantly higher variance of pore diameter, lower circularity, and higher eccentricity compared to the samples prepared with the other two conditions (Figure 2E-G). Taken together, this data highlights that the microarchitecture was less homogeneous in pore diameter across the structures when prepared with a *T*_f_ of −80 °C with pores characterized by a more elongated as well as a more irregular profile. In agreement with previous works,^49^ both findings are characteristic of quenching (i.e., rapid-freezing), and it could be concluded that at *T*_f_ of −80 °C the freezing process occurred at a dramatically higher cooling rate causing a preferential direction in the heat transfer and the subsequent generation of a more heterogeneous pore size distribution as well as in a preferential direction despite the use of a standard mold.

From this we can conclude that both −20 °C and −40 °C generated more isotropic samples with −40 °C also yielding a smaller pore size. Because of the high regularity of the pore geometry and the smaller pore size, the *T*_f_ −40 °C was identified as the most suitable candidate for a comparison with the aligned scaffolds, and it was adopted for all subsequent experiments.

#### 2.1.2 Incorporation of PDMS Elastomeric Matrices Generates Biphasic EAS

The 3D scaffolds generated with PEDOT:PSS-GOPS have already been shown to be stable, insomuch that they have been used in wet applications such as tissue engineering.^16,32^ However, because this material is nonelastomeric and fragile,^23^ repetitive mechanical deformation, which is typical of physiological applications, can eventually alter and deteriorate their stuctures. Such a change in morphology drives a modification of the piezoresistivity over time, and therefore it is not compatibile with the development of long-lasting piezoresistive sensors.

Others have reported the incorporation of an elastomeric component at the moment of material processing as its presence can allow for more suitable mechanical properties.^37^ However, such approaches can impact the electrical properties of the conductive polymers and they do not allow for the processing of different microarchitectures with the same simplicity as freeze-drying. One common approach to incorporate a conductive material and an elastomer is the functionalization of a porous matrix with a thin coating of a conductive polymer or nanoparticles; an example is a sensor described by Yang et al.^3^ who designed a porous PDMS containing a micropyramid structure adhered to and spaced upon a soft EcoFlex matrix and coated with AgNW.

**Figure 3:**
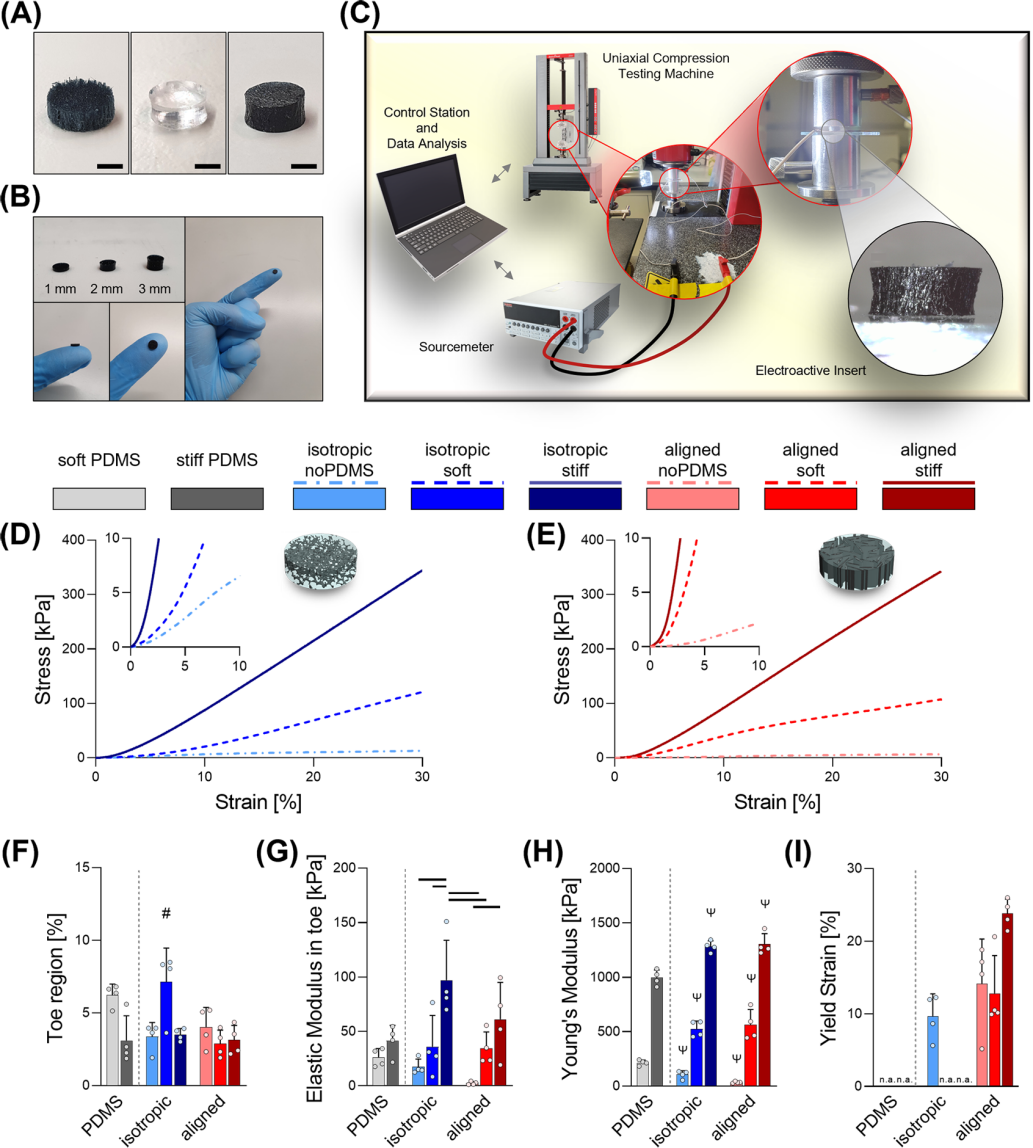
Manufacturing and testing of biphasic EAS. (A) Pictures of a PEDOT:PSS-GOPS scaffold (left), a pure PDMS sample (middle), and a biphasic EAS (right). (B) Picture of three biphasic EAS cut at different thicknesses (i.e., 1/2/3 mm) and pictures of a 1 mm thick sample handled by the user. (C) Schematic of the piezoresistivity testing setup. (D,E) Mean stress–strain curves for scaffolds and EAS embedded with both soft and stiff elastomers with isotropic (D) and aligned morphology (E). Insets show details of the regions between 0 and 10 kPa stress and 0–10% strain. (F–I) Quantification of different mechanical parameters (*n* = 4): (F) extension of the toe region, (G) elastic modulus of the toe region, (H) Young’s modulus, and (I) yield strain, where “n.a.” indicates that the yield strain was not reached in the range of deformation applied in the test. Scale bars: *A* = 1 mm. Bar graphs demonstrate the mean with error bars representing standard deviation. Data values are presented as associated points. PDMS-only groups were not included in the statistical analysis. Line represents statistical significance (*p* < 0.05) between indicated groups, # represents statistical significance (*p* < 0.05) between the indicated group and all other groups, ψ represents statistical significance (*p* < 0.05) between the indicated group, and all other groups excluded the one with same stiffness. Statistical analysis was performed using two-way ANOVA with Tukey’s post-hoc test.

Here, we describe a rarely reported alternative strategy,^50^ whereby we investigated the combination of an elastomeric matrix with conductive PEDOT:PSS scaffolds (Figure 3A). We chose standard PDMS and engineered two contrasting stiffnesses by combining Sylgard 184 and Sylgard 527 at different ratios.^41^ Pure Sylgard 184 to generated a “stiff” elastormeric matrix and a mix of 184 and 527 at ratio 1:5 for the “soft” one. This process can be expanded by adjusting the ratio between the two compounds in order to obtain stiffnesses between 50 kPa and 1.5 MPa.^41^ Once the PEDOT:PSS-GOPS scaffold and the PDMS matrix were combined, they were fashioned into a cylindrical morphology using standard 5 mm biopsy punches and cut in slices ranging from the hundreds of micrometers up to 3 mm so as to suit diverse sensor designs (Figure 3B).

We have previously reported this range of PEDOT:PSS-GOPS scaffolds with values of porosity as high as 95%.^16^ With the addition of the PDMS infusion step, we ensured that all empty space within the scaffold porous structure were perfused by this insulant elastomer. The final product is a construct primarily composed of the PDMS insulant material that maintains the original electroconductive characteristic of the PEDOT:PSS-GOPS scaffold within.

### 2.2 Piezoresistivity Analysis in Ramp Condition

The setup adopted for electromechanical analysis of the EAS under compressive deformation is reported in Figures 3C and S2. From the stress–strain curves one can immediately observe the diverse mechanical responses of these constructs (Figures 3D,E and S3).

Analysis of the strain extension of the toe region reveals a significant increase for the “isotropic soft” group compared to all others (Figures 3F). However, the more intriguing properties are those evidenced by the Young’s moduli in both the toe and the linear regions (Figure 3). As expected, the PEDOT:PSS-GOPS scaffolds with “noPDMS” exhibited significantly softer structures with the aligned geometry showing less rigidity than the isotropic one in accordance with our previous findings.^16^ For the Young’s moduli of both toe and elastic regions, a synergistic behavior of the scaffold and the elastomeric matrix was evident, showing stiffer responses for the biphasic EAS groups than for the PDMS alone. In addition, no significant differences were found between isotropic or aligned samples when stiff or soft PDMS were used.

As shown in Figure 3I, paying attention to the yield strain highlights that the introduction of a PDMS matrix increases the range of elastic deformation that can be applied to the EAS, shifting from approximately 9.6% up to beyond 30% for the isotropic group and from 14% up to 23.8% for the anisotropic one. This is an important finding, demonstrating that the mechanical behavior of the elastomeric materials constituting the matrix phase dominates the mechanical behavior.

**Figure 4:**
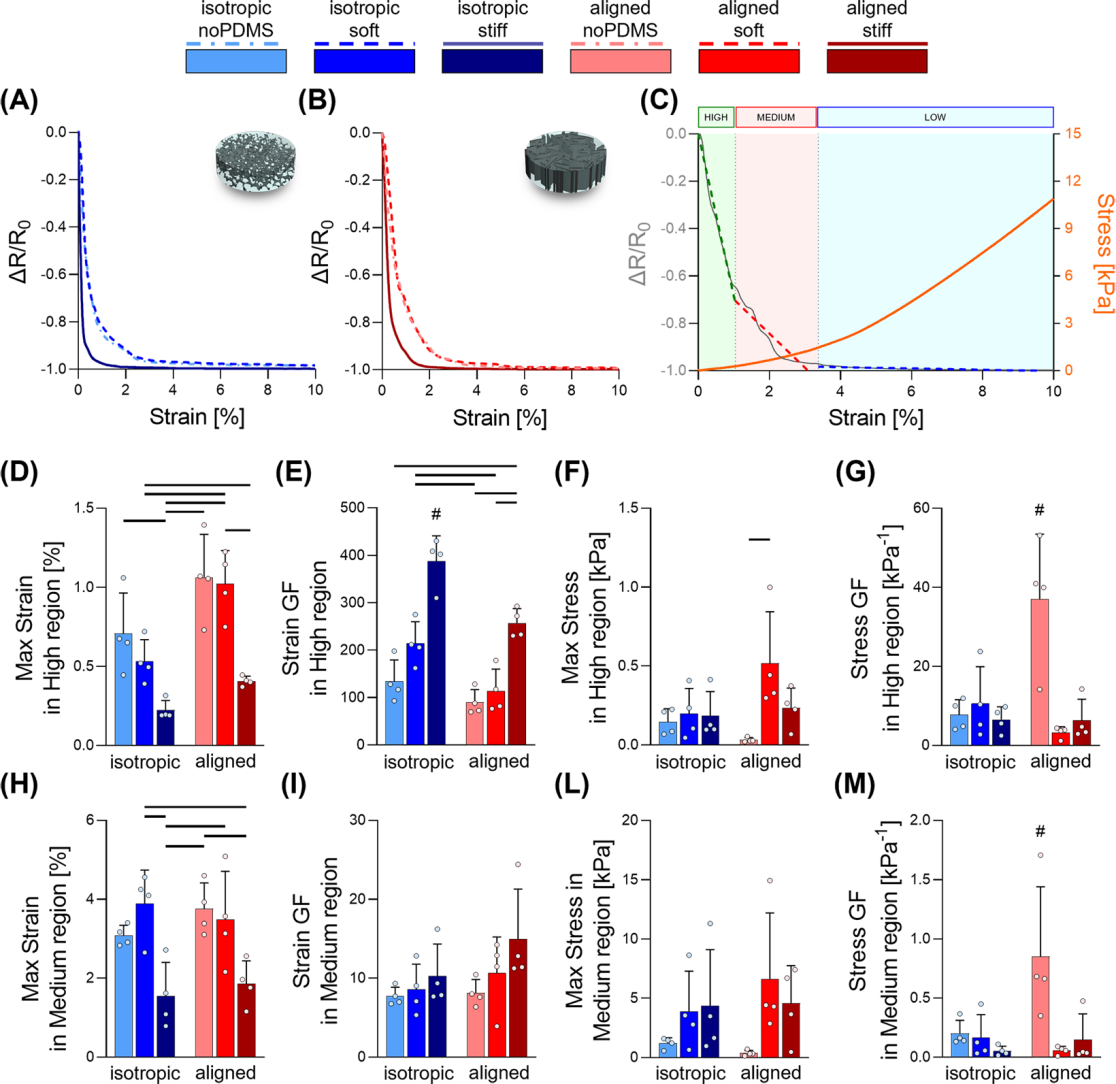
Analysis of the piezoresistive response of the biphasic EAS in the ramp phase. (A,B) Mean curves of the variation of resistivity over strain for scaffolds and biphasic EAS embedded with both soft and stiff elastomers with isotropic (A) and aligned morphology (B). (C) Representative graph of the identification of high, medium, and low output regions in the curves of the variation of resistivity over strain. (D–M) Quantification of different piezoresistivity parameters for the high region (D–G) and the medium region (H–M) (*n* = 4): (D,H) maximum strain in the region, (E,I) strain gauge factor in the region, (F,L) maximum strain in the region, and (G,M) stress gauge factor in the region. Bar graphs demonstrate the mean with error bars representing standard deviation. Data values are presented as associated points. Line represents statistical significance (*p* < 0.05) between indicated groups, # represents statistical significance (*p* < 0.05) between the indicated group and all other groups. Statistical analysis was performed using two-way ANOVA with Tukey’s posthoc test.

Results of the piezoresistive characterization when subjected to a ramp phase are reported in Figure 4. Undeformed biphasic EAS exhibit conductivity values that do not differ within the “isotropic” groups, while a decrease was observed for the “aligned stiff” compared to the “aligned noPDMS” (Figure S4). Figure 4A,B illustrates the profiles of the average relative change in resistance over strain, whereby it is evident that in both isotropic and aligned conformations, the stiff matrices provide a more sudden response rather than the soft ones that are more similar to the noPDMS groups. As reported in other studies,^43^ we identified three strain regions in which the gauge factor can be taken as constant, namely a “high” region with the biggest resistance variation, a “medium” region where a variation could still be perceived, and finally a “low” region in which the variation is almost negligible (Figure 4C).

Figure 4D–G illustrates a series of key parameters that were quantified from the analysis of the curves, where the high sensitivity region was characterized by its extension range as maximum strain and maximum stress, as well as by the relative strain-dependent and stress-dependent gauge factors. Soft and noPDMS groups highlighted a wider range of deformation, while a significant increase between “aligned” or isotropic geometries was reported within the soft groups. The strain-dependent gauge factor followed an opposite response, and the highest gauge factors were reported for the “isotropic stiff” and the aligned stiff groups (387.8 ± 53.2 and 257 ± 30.4, respectively). The maximum stress range detected in this region were consistent among most of the groups tested with the aligned noPMDS condition, showing a significant increase compared to the “aligned soft” group. As for the analysis of the strain, the stress-dependent gauge factor decreased when the stress limit increased. The “aligned noPDMS” reached values as high as 37 ± 16.4 kPa^–1^, significantly higher than all other groups which reported sensitivities between 3.3 and 10.6 kPa^–1^.

In Figure 4H–M, the same parameters were quantified for the medium sensitivity regions where trends similar to the high sensitivity region were observed. The strain-dependent gauge factor showed an increasing trend with the augmented stiffness of the EAS, reaching values of 10.3 ± 4 and 15 ± 6.3 for the isotropic stiff and aligned stiff groups, respectively. As for the high sensitivity region, the highest stress-dependent gauge factor was obtained for the aligned noPDMS group with 0.85 ± 0.59 kPa^–1^.

Most works on compressive sensors report a significantly inferior strain-dependent gauge factor such as 26.07 for a polyurethane-based cracked cellulose silver nanowire (strain range 0–0.6%)^51^ and 2.1 for a graphene oxide/polypyrrole polyurethane sponge (strain range 0–40%).^29^ Few reports describe strain-dependent sensitivities in this magnitude range^43^ with the only exception being that of a graphene-putty sensor.^52^ However, it is important to consider that the biphasic EAS we are presenting here has a reduced range of deformation in the high sensitivity region, with potential limitations on its applications. Our work represents a significant development for the consideration of PEDOT:PSS within a compressive sensor. To date, PEDOT:PSS based materials have been successfully employed for their piezoresistive properties, but primarily as a coating with the examples of the PDMS-based pressure sensor with micropyramid array structure and a PEDOT:PSS/polyurethane coating produced by Chong et al.,^53^ and the PEDOT:PSS/melamine sponge produced via dip-coating and freeze-drying procedure by Ding et al.^18^

### 2.3 Piezoresistivity in Dynamic Condition

Viscoelasticity is a common feature of polymeric materials, where the material exhibits a combination of elastic and viscous properties of mechanical recovery without energy dissipation and the capacity to dissipate energy, respectively.^54^ Under uniaxial strain, the combination of these two behaviors often results in hysteresis, where a difference in stress response occurs between the load and unload stage. Materials with viscoelastic properties respond to dynamic loading in a time-dependent manner, where both strain rate and amount of strain applied will have an impact on the mechanical behavior.

Hysteresis error is a primary source of uncertainty in strain measurements^55^ and a common challenge of stretchable polymer-based sensors as it also affects electrical properties which are closely correlated,^7^ producing a nonlinear resistance output. Stress dissipation over dynamic cyclic loading is another property commonly seen in viscoelastic materials such as polymers, where a constant applied strain results in the materials gradually absorbing deformation and reducing stress to a final steady value.^8^

**Figure 5:**
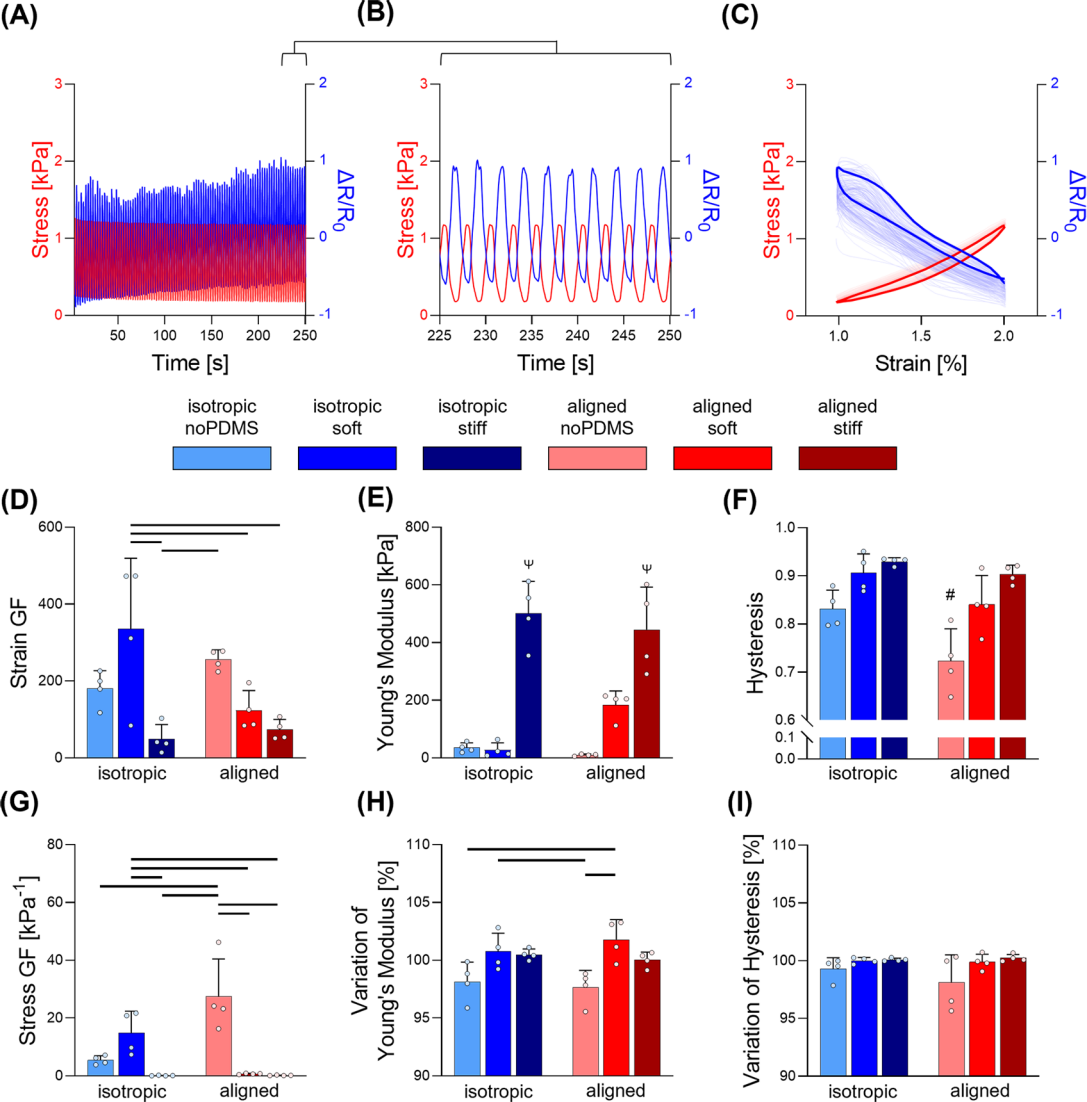
Analysis of the piezoresistive response of the biphasic EAS in the cyclic phase. (A–C) Representative curves of the stress and resistivity variation over time for the 100 cycles (A), the last 10 cycles (B), and over strain (C). (D–I) Quantification of different piezoresistivity parameters over the cyclic stimulation (*n* = 4): (D) average strain gauge factor of the last 10 cycles, (E) average stress gauge factor of the last 10 cycles, (F) average Young’s modulus of the last 10 cycles, (G) average mechanical hysteresis of the last 10 cycles, (H) variation of the average Young’s modulus between the cycles 21–30 and the last 10, and (I) variation of the average Young’s modulus between the cycles 21–30 and the last 10. Bar graphs demonstrate the mean with error bars representing standard deviation. Data values are presented as associated points. Line represents statistical significance (*p* < 0.05) between indicated groups, # represents statistical significance (*p* < 0.05) between the indicated group and all other groups, ψ represents statistical significance (*p* < 0.05) between the indicated group and all other groups excluded the one with same stiffness. Statistical analysis was performed using two-way ANOVA with Tukey’s posthoc test.

To understand the performance of the EAS under dynamic movements typical of muscoloskeletal movement, we examined the piezoresistive response during a cyclic regime and constant frequency. Mechanical and electrical variations were quantified in the range of deformation 1–2% and their variation over time was quantified (Figure 5A–C).

Measurements in terms of the average data acquired in the last 10 cycles of the tests are reported in Figure 5D–G. The applied deformation corresponded to very diverse average stress ranges: 0.07–0.44 kPa for “isotropic noPDMS”, 0.14–0.43 kPa for isotropic soft, 1.14–6.32 kPa for isotropic stiff, 0.006–0.11 kPa for aligned noPDMS, 0.38–2.31 kPa for aligned soft, and 0.76–5.39 kPa for aligned stiff.

Compared to the results obtained in the ramp test, some differences can be observed in the quantification of the mechanical and piezoresistive properties, because of the adoption of a more limited strain range. In particular, it is evident that the isotropic soft group exhibited less rigid mechanics and how this lead to higher strain-dependent and stress-dependent gauge factors compared to the isotropic stiff. Significantly higher values for both these parameters were also reported for the aligned noPDMS group.

A significantly lower elastic hysteresis was present in the aligned noPDMS group, suggesting that a range of deformation as small as 2% already caused a less elastic response of the material.

We quantified the variation of stiffness and hysteresis over the cycles and similar trend for both isotropic and aligned geometries, with the two noPDMS groups showing significant higher reduction of mechanical properties over time. This confirmed how the presence of elastomeric matrices, either soft or stiff, paves the way for the use of biphasic EAS for long-term applications. Indeed, this family of biphasic EAS can prevent the loss of mechanical stability and the consequent deteriation of piezoresistivity that would reduce of the sensor performance by structural failure. As it can be observed in Figure 5.A, the resistance variation is subjected to a quasi-linear drift over time, a phenomenon that has to be considered during the signal processing phase and that is a common feature to several porous 3D sensors.^45,56^

### 2.4 Validation of a Piezoresistive Sensor Prototype

**Figure 6:**
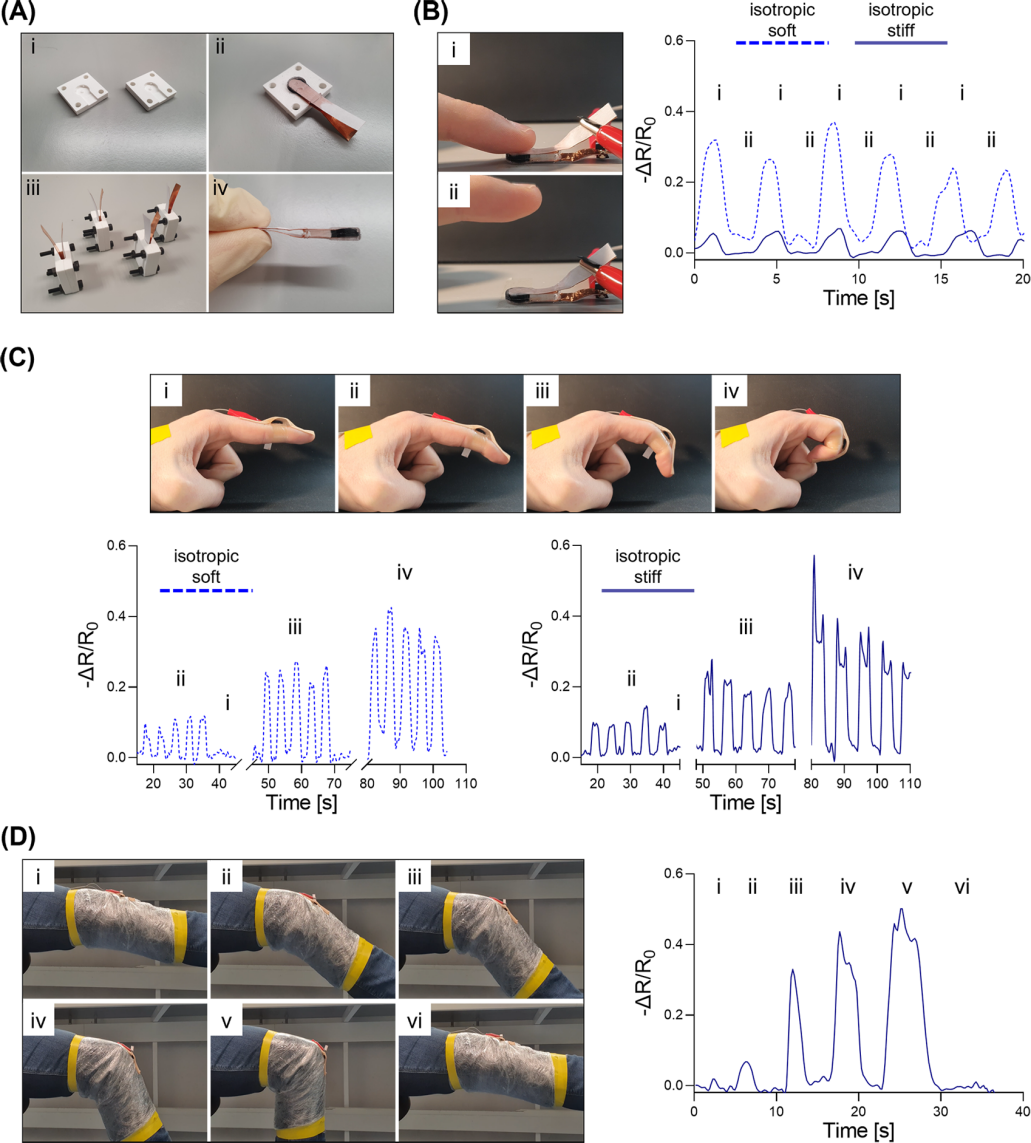
Fabrication of a piezoresistive sensor prototype. (A) A custom-made mold able to fit a biphasic EAS with two copper tapes as electrodes (i/ii). Once closed, PDMS can be casted in the mold, and sensors obtained (iii/iv). (B) Finger tapping sequences for sensors fabricated using either isotropic soft or isotropic stiff inserts. (C) Finger bending sequence for three ranges of motion, with either isotropic soft or isotropic stiff sensors. (D) Knee bending sequence for three ranges of motion with isotropic stiff sensor.

Prototypes of piezoresistive sensors were manufactured utilizing inserts based on the isotropic soft and isotropic stiff PEDOT:PSS platforms developed in this study (sequence of assembly and manufacture is illustrated in Figure S5). Briefly, a custom-made mold was designed to incapsulate the insert together with two electrodes consisting of copper tapes in one complete piece (Figure 6A). This sensor can be applied directly to the skin and secured with standard bandages. In this validation, we focused on standard limb movements such as finger and knee bending, during which signals were detected with a sourcemeter and processed with a Matlab script.

Results in previous sections of this paper have already suggested that an isotropic conductive scaffold backbone has improved performance in comparison to an anisotropic one, while varying magnitudes of sensitivity are achieved depending on elastomeric stiffness. Figure 6B shows that inserts of different stiffnesses responded differently even when subjected to the same pressure. When stimulated with a repetitive finger tapping exercise, the isotropic soft insert was able to generate a higher output signal than the isotropic stiff counterpart. This confirms that the biphasic EAS can indeed be modified in their stiffness to match the required sensitivity for a specific application.

To establish the capacity of these sensors to detect range of motion, we repeated a finger bending test at different magnitudes of bending extension using either isotropic soft or isotropic stiff inserts Figure 6C. From these results, the output signals of the sensors were proportional to the degree of bending applied with no remarkable differences between the two samples of different stiffnesses. This strongly agrees with our central hypothesis, whereby to similar deformation of the sensor should correspond a comparable resistance variation, independently from the stiffness of the sensor adopted. In particular, for all three ranges of motion that were investigated (i.e, regions i, ii, and iii) both isotropic soft and isotropic stiff sensors worked within the medium sensitivity region, where their strain-dependent gauge factor were at comparative levels (Figure 4C). Similarly, an isotropic stiff platform could detect different ranges of knee bending Figure 6D. Clear repetitive patterns were also observed when using this sensor prototype for swallowing and tight muscle tensing actions (Figure S6).

Although two contrasting stiffnesses were investigated in this study, one could fine-tune the mechanics of the matrix quite easily by varying the ratio between the two types of PDMS. Taken together, we have clearly demonstrated with the combination of microarchitecture design and filler stiffnesses that sensors can be optimized to operate with high sensitivity in the typical range of each specific application.

For the validation of this family of biphasic EAS, we adopted PEDOT:PSS-GOPS scaffolds that were characterized by relatively high stiffness and low conductivity. While the analysis here focuses on the impact of mechanical properties on resistance properties, other options to gain further information and output from this piezoresistive sensor in the future could include the investigation of frequency dependent AC conductivity as reported by others.^57^ Our group has previously demonstrated how both the mechanical and electrical properties of this material can be affected and ameliorated by crystallization post-treatment with sulfuric acid^16^ or how such properties can be influenced by the use of different cross-linker as poly(ethylene glycol)diglycidyl ether.^15^ Such approaches could be used to modify this platform even further. The use of these materials as electroconductive scaffolds combined with a soft elastomer could lead to EAS characterized by wider high sensitivity strain range and possibly able to detect even lower stresses.

## 3 Conclusion

In the development of piezoresistive components for wearable sensors, conductive fillers such as graphene or silver nanowires are most frequently being used to achieve superior sensitivity but they are associated with significant drawbacks such as high cost, toxicity, and complex fabrication methods which limit their scalability and accessibility. On the other hand, PEDOT:PSS is an intrinsically conductive polymer that can easily and safely be handled and processed in a laboratory and which exhibits piezoresistive properties. Also, as this compound is often provided as a dispersion in water the morphology of the final constructs can be controlled by use of different freeze-drying parameters during fabrication.

Here, we presented the fabrication of a range of PEDOT:PSS-based porous structures and their combination with a PDMS elastomer for the obtainment of EAS. When benchmarking our approach against previous reports on the use of conductive polymers as coatings upon a porous material or mixed as a secondary component in a slurry containing an elastomer, we instead fabricate 3D PEDOT:PSS-GOPS scaffolds and afterward infuse with elastomer to obtain biphasic EAS. Mechanical and piezoresistive properties of these EAS were characterized, providing detailed sets of results on their response in ramp and cyclic conditions, as well as on their performance as piezoresistive sensors.

It was demonstrated that an isotropic electroconductive scaffold provides better overall mechanics with an extended toe region and lower hysteresis, and when combined with an elastomeric matrix their yield point was extended up to beyond 30% strain, demonstrating that these constructs can be used for a wide range of deformation without progressing into plastic transition. Significantly, we demonstrated that the presence of a stiffer PDMS filling yields a reduction in the range of high sensitivity, however it also caused a profound increase in strain-dependent gauge factor with values up to almost 400 for the isotropic group, that few reports were able to reach. Conversely, the use of a softer matrix yielded lower sensitivity but within an extended strain range. We can conclude that we optimized a method for cheap and safe fabrication of piezoresistive sensor with adaptable sensitivity range and high strain sensitivity. According to the final application of the piezoresistive sensor, namely, the strain or stress range and the required sensitivity, a different combination of microarchitecture and elastomer stiffness can be used for achieving the best output.

## 4 Experimental Methods

### Fabrication of 3D PEDOT:PSS Sponges

PEDOT:PSS 1.3 wt % dispersion in water and GOPS were purchased from Sigma-Aldrich (Sigma-Aldrich, Ireland). Preparation of blends with a 3 v/v% GOPS component and fabrication of sponges through lyophilization were performed similarly to how it is described in our previous study.^16^ Three different freezing temperature were adopted to influence the shape and size of the porous architecture, namely, −20 °C, −40 °C, and −80 °C, while anisotropy was guided by freezing the material at −40 °C inside a custom-made mold (Figure S1). Briefly, molds already containing solutions at room temperature were added to a precooled freezing chamber. This process indirectly allowed for the application of three different cooling rates given by the three different thermal gaps, ultimately affecting the freezing dynamic of the sponge-like scaffolds. Dry sponges underwent annealing treatment in a vacuum oven at 140 °C for 1 h; afterward, they were subjected to multiple washings using deionized water, and finally a second lyophilization process was carried out to fully dry the materials.

### Morphology of 3D PEDOT:PSS Sponges

X-ray microtomography (μCT, Scanco, Switzerland) was employed to allow for a macroscopic 3D reconstruction of scaffold morphology. Qualitative high-magnification investigation was also performed by scanning electron microscopy (SEM). Briefly, 3D porous scaffolds were mounted on aluminum stubs with a conductive carbon tape (Ted Pella, U.S.A.), and a gold–palladium layer of approximately 5 nm was sputter coated on the sample surface. Specimens were observed using a Zeiss SUPRA 40 field emission SEM (Zeiss, Germany) with a 5 kV electron beam.

Sections of the scaffolds were imaged with a ScanScope (Aperio Technologies Inc., U.S.A.) after a multistep process of agarose and wax embedding as previously reported.^16^ Quantification of pore size, pore circularity, and pore eccentricity was then performed with a custom-made Matlab script.

### Infusion of Elastomeric Fillers

Two formulations of polydimethylsiloxane (PDMS, Sylgard 184 and Sylgard 527) were combined to achieve different stiffness. Both products were individually prepared according to manufacturer instruction, whereby a stiff elastomer was obtained by pure Sylgard 184, while a soft one was achieved by mixing the two products with a 1:5 ratio (184:527). PEDOT:PSS-GOPS sponges were then placed into astandard plastic molds and PDMS was added up to fully cover the scaffolds and multiple cycles of vacuum were performed to allow for the elastomers to infiltrate throughout the whole scaffold. Constructs were cured at 60 °C for 2 h to allow for PDMS polymerization and afterward they were embedded into an agarose solution to allow easier cutting into thin slices of desired thickness ranging from 0.5 to 3 mm via a vibratome (VT 1200S, Leica, Germany). Slices were finally cut with standard cylindrical biopsy punches to obtain thin circular samples with fixed diameter.

### Investigation into the Electromechanical Response

To perform the electromechanical analysis, we developed a custom-made setup similar to what has been reported elsewhere.^58^ A Zwick Roell twin column universal testing machine (ZO50, Zwick/Roell) with a 10 N load cell was used to apply static or cyclic strain to the EAS while a sourcemeter Keithley 2400 (Tektronix, U.S.A.), controlled via python software was used to measure the electrical resistance of the EAS as a function of time (Figure 3C).

The EAS was placed on the bottom electrode, while the top platen was gradually lowered closer to the sample in increments of 10 μm using the machine’s automatic adjustment feature until full surface contact was established. EAS with 2 mm thickness were used for the analysis.

A uniaxial cyclic and a ramp compression test were performed in a consecutive sequence with a break of 60 s in between them. The testing sequence is represented by strain variation over time during the test and can be seen in Figure S2. The cyclic test consisted of 100 cycles applied to the sample between 1% and 2% strain at a compression rate of 1% s^–1^ or 0.5 Hz. The ramp test consisted of a single cycle where compressive strain was applied up to 30% of the sample height, at a compression rate of 0.1% s^–1^. During the 60 s hold period between tests, strain returned to the preload value.

Subsequently, recordings from both the Zwick and Keithley were transferred to the control center and the two data sets were merged at corresponding time intervals by using specialized python code to align the starting timestamp of both systems. Finally, any remaining electrical noise was filtered in Matlab using a built-in “hampel” filter followed by a third order “Savizky-Golay” smoothing filter and a second order low-pass “Butterworth” filter.

A series of custom-made Matlab scripts were written so to extrapolate multiple measurements from this single cyclic-ramp combined test.

From the analysis of the cyclic phase, the dynamic strain-dependent, and stress-dependent gauge factors, the mechanical hysteresis, the variances of the stiffness and of the sensitivity throughout the cycles were extrapolated.

From the ramp phase, it was possible to define mechanical parameters such as toe region range, toe region stiffness, Young’s modulus, and yield strain where present. The scripts allowed one to identify three regions in the electromechanical signals and to derive measurements of the ranges of these regions in terms of strain [%] and stress [kPa] and the corresponding strain- and stress- gauge factors.

### Sensor Fabrication and Testing

To investigate the sensor capability to detect and monitor physiological signals, a proof-of-concept sensor was built and tested on a range of physiological motions such as finger and knee bending, swallowing, and muscle tensing. The sensor was fabricated taking inspiration from previously described methods.^59^ A schematic of the components is reported in Figure 1B, while the assembly sequence can be observed in Figure S5 with two copper tapes working as electrodes and being connected to the two sides of either isotropic soft or isotropic stiff inserts in a sandwich-like structure. We designed a custom-made mold that could fit 3 mm think biphasic EAS or scaffolds, and once sealed it allowed for PDMS casting. After PDMS cross-linking, the system was opened, and the sensor prototype harvested. This method allowed for high reproducibility of the sensor fabrication and performances. Figure 6A shows a representation of this prototype. Once the sensor was applied to the skin through the use of a flexible adhesive tape, the electrical response was continuously monitored using the Keithley 2400 sourcemeter.

## Supporting Information Available

(Figure S1) Schematic showing the custom-made mold; (Figure S2) sequence of testing; (Figure S3) mean stress–strain curves for *soft* and *stiff* elastomers; (Figure S4) quantification of conductivity at 0% strain; (Figure S5) assembly sequence of the piezoresistive sensor prototype; (Figure S6) sensor proof-of-concept signals: swallowing and thigh muscle tensing (PDF)

## ABBREVIATIONS

3D: three-dimensional
EAS: electroactive sponges
GOPS: glycidoxipropyl-trimethoxysilane
PDMS: polydimethylsiloxane
PEDOT:PSS: poly(3,4-ethylenedioxythiophene):polystyrenesulfonate
SEM: Scanning electron microscopy
