# Supplementary material for: Beyond Chemistry: Tailoring Stiffness and Microarchitecture to Engineer Highly Sensitive Biphasic Elastomeric Piezoresistive Sensors
Source: ACS Appl Mater Interfaces. 2022 Apr 22;14(17):19265–77. doi: 10.1021/acsami.2c04673 (PMC9073843; doi:10.1021/acsami.2c04673)
Supplement: Supplementary file 1 — am2c04673_si_001.pdf [file am2c04673_si_001.pdf]

Supporting Information

Beyond Chemistry: Tailoring Stiffness and Microarchitecture to Engineer Highly Sensitive Biphasic Elastomeric Piezoresistive Sensors.

*Matteo Solazzo [1,2], Linette Hartzell [1,2], Ciara O'Farrell [1,2], Michael G. Monaghan [1,2,3,4] **

1. Department of Mechanical, Manufacturing and Biomedical Engineering, Trinity College Dublin, Dublin 2, Ireland.
2. Trinity Centre for Biomedical Engineering, Trinity College Dublin, Dublin 2, Ireland.
3. Advance Materials and BioEngineering Research (AMBER) Centre at Trinity College Dublin and the Royal College of Surgeons in Ireland, Dublin 2, Ireland.
4. CÚRAM, Centre for Research in Medical Devices, National University of Ireland, Galway, Newcastle Road, H91 W2TY Galway, Ireland

* Prof. M. G. Monaghan

Trinity Centre for Biomedical Engineering, Trinity Biomedical Sciences Institute, Trinity College Dublin, 152–160 Pearse Street, Dublin 2, Ireland. E-mail: monaghmi@tcd.ie

Keywords: piezoresistive sensors, conductive polymers, pedot:pss, strain sensors, conductive elastomer

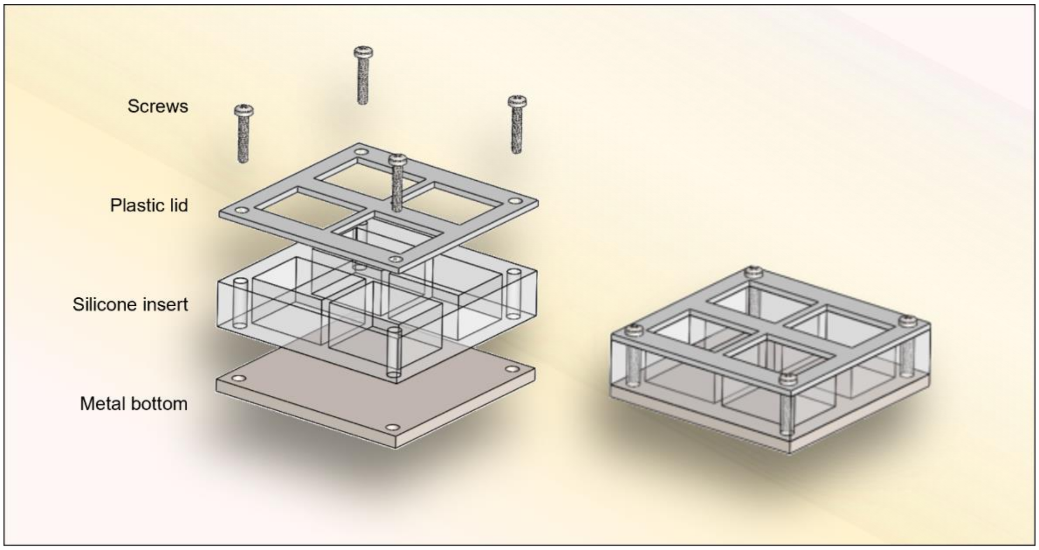

Supporting Information 1. Schematic showing the custom-made mold that was used for the manufacturing of aligned PEDOT:PSS-GOPS scaffolds.

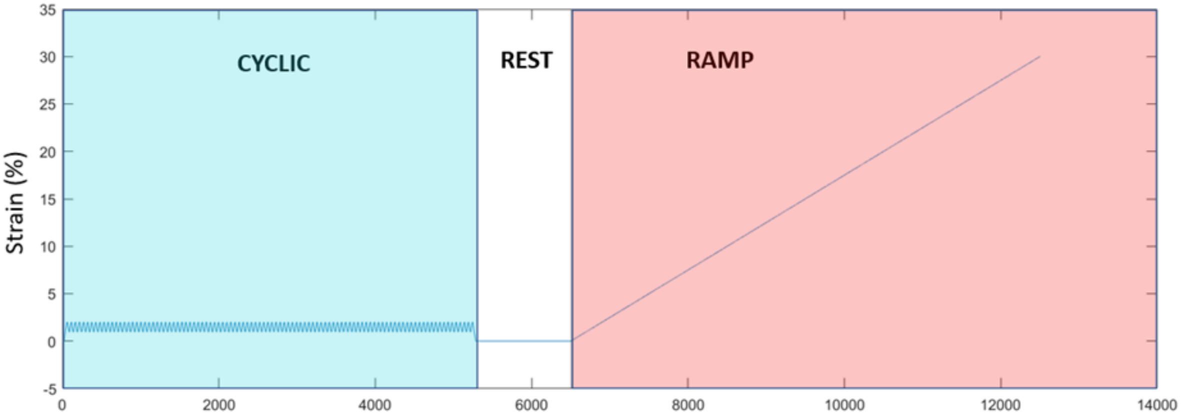

Supporting Information 2. Sequence of testing. Dynamic Cyclic Testing: 100 cycles, 1-2% strain, 1%/s. Rest – at preload, 60 seconds. Ramp Testing – 1 cycle, 0-30% strain, 0.1%/s.

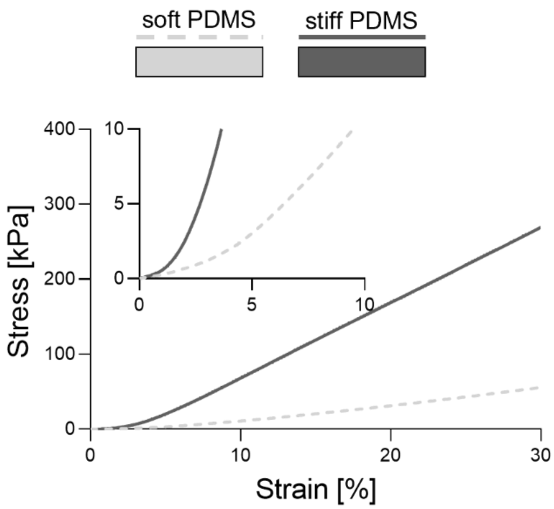

Supporting Information 3. Mean stress-strain curves for soft and stiff elastomers.

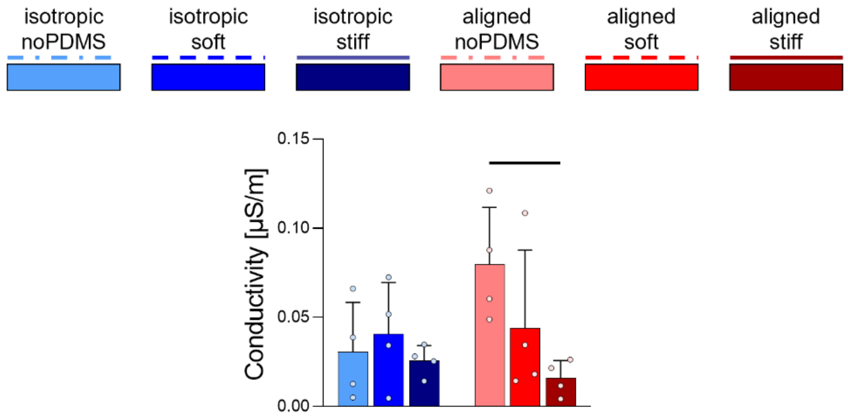

Supporting Information 4. Quantification of conductivity at 0% strain. Bar graphs demonstrate the mean with error bars representing standard deviation. Data values are presented as associated points. Line represents statistical significance ($p<0.05$) between indicated groups using two-way ANOVA with Tukey's post-hoc test.

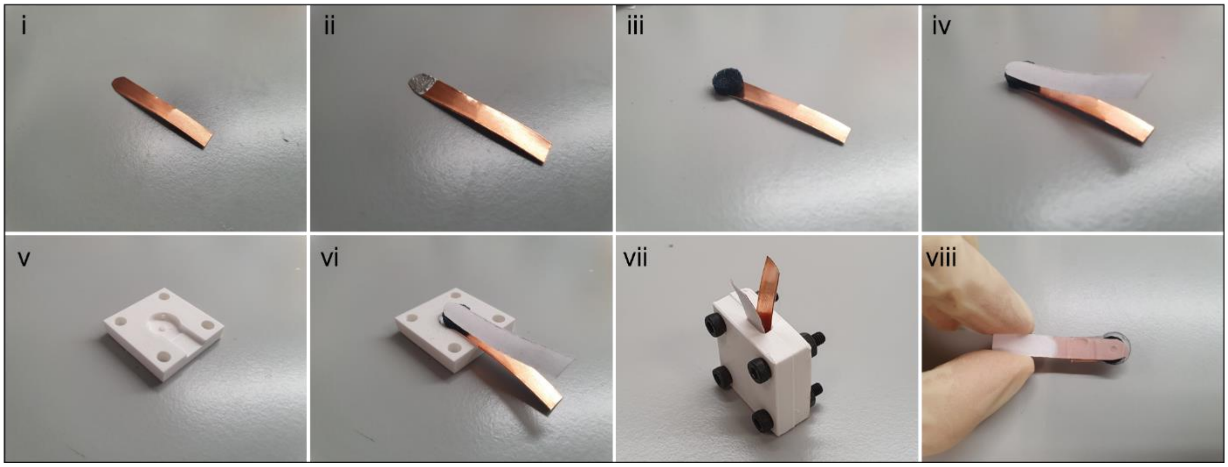

Supporting Information 5. Assembly sequence of the piezoresistive sensor prototype. (i-ii) Silver paint is applied to a copper tape. (iii) A 3 mm thick biphasic EAS is positioned on the silver paint. (iv) More silver paint is applied to a second copper tape, that is then positioned on top of the biphasic EAS. (v) This assembly is positioned on the bottom half of a custom-made mold. (vii) A second half of the custom-made mold closes the apparatus, and it is secured with 4 screws. The apparatus is ready for PDMS casting. (viii) After PDMS crosslinking, the apparatus can be opened, and the sensor prototype obtained.

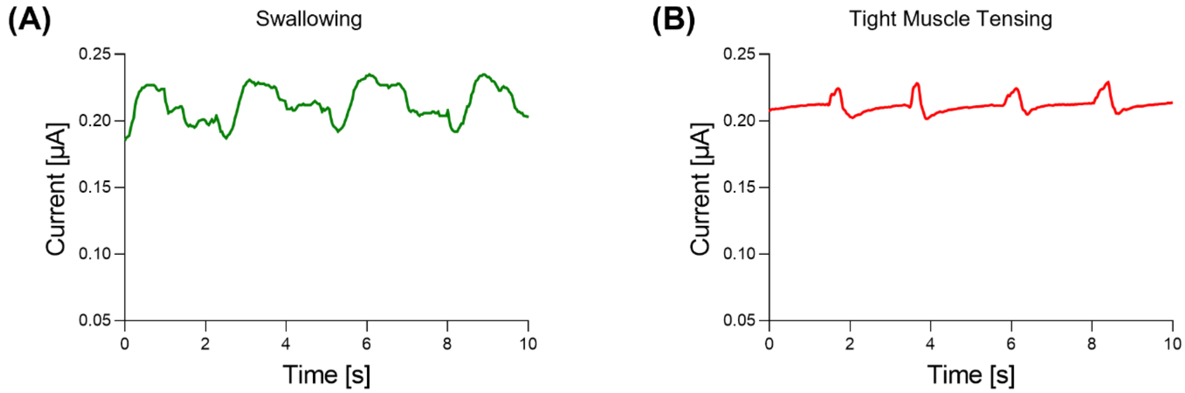

Supporting Information 6. Sensor proof-of-concept signals: **(A)** swallowing and **(B)** thigh muscle tensing.
